# Effect of fermented feed material on growth performance and health parameters of Yorkshire/Norwegian Landrace/Duroc (J/NL/D) F2 crossbred fattening piglets

**DOI:** 10.3389/fvets.2026.1839557

**Published:** 2026-07-08

**Authors:** Sarunas Badaras, Vytaute Starkute, Ernestas Mockus, Modestas Ruzauskas, Dovile Klupsaite, Laurynas Vadopalas, Elena Bartkiene

**Affiliations:** 1 Institute of Animal Rearing Technologies, Faculty of Animal Sciences, Lithuanian University of Health Sciences, Kaunas, Lithuania; 2 Department of Food Safety and Quality, Faculty of Veterinary Medicine, Lithuanian University of Health Sciences, Kaunas, Lithuania; 3 Department of Anatomy and Physiology, Faculty of Veterinary Medicine, Lithuanian University of Health Sciences, Kaunas, Lithuania; 4 Institute of Microbiology and Virology, Faculty of Veterinary Medicine, Lithuanian University of Health Sciences, Kaunas, Lithuania

**Keywords:** blood parameters, body weight, feces bacterial composition, feces volatile compounds, feed conversion ratio, immunoglobulins

## Abstract

Nutritional approaches to enhancing pig health and production efficiency remain an important area of research. In this study, the effect of supplementing the diet of Yorkshire/Norwegian Landrace/Duroc (J/NL/D) F2 crossbred fattening piglets with *Lactobacillus* spp.-fermented feed material (Lb.-FM) was investigated. Growth performance and health parameters were evaluated, including body weight (BW), feed conversion ratio (FCR), plasma concentrations of immunoglobulins (IgA, IgM, and IgG), the activities of aspartate aminotransferase (AST), and alanine aminotransferase (ALT). Additionally, fecal parameters were analyzed, including pH, dry matter (DM) content, texture hardness, lactic acid bacteria (LAB), total enterobacteria counts (TEC), yeast and mold (Y and M) counts, and fecal microbial metataxonomic profiles. Furthermore, fecal volatile organic compound (VOC) profiles were analyzed as sensitive chemical markers of nutrient metabolism. The piglets were reared on a group farm operating under a multi-site production system. Piglets were obtained from the “Vyturys” Agricultural Company farm (Lithuania) and, after weaning at 21 days of age, weighing an average of 6.3 kg, were transported to the “Kontvainiai” Agricultural Company farm (Lithuania) for further fattening. The experiment began with 21-day-old piglets (after weaning) and lasted 40 days (until the 61st day of the piglets’ lives). The animals were divided into two groups: 175 piglets in the control (C) group, which received a complete commercial feed, and 177 piglets in the Lb.-FM group, which received Lb.-FM with *Lactiplantibacillus plantarum*, *Lacticaseibacillus casei, Latilactobacillus curvatus*, and *Lacticaseibacillus paracasei*. The results showed that the inclusion of Lb.-FM in piglet diets improved FCR, suggesting enhanced nutrient utilization. Additionally, Lb.-FM did not significantly affect growth performance, blood parameters, fecal physicochemical properties, microbial counts, or faecal VOC profiles, confirming its safety and suitability. It also positively influenced gut microbiota composition without disrupting the core microbial balance. Overall, age-related changes had a greater impact than diet on most analyzed parameters, indicating that the benefits of fermented feed are more functional than quantitative. In conclusion, the findings of this study show that fermented feed containing *Lb*. *plantarum*, *Lb. casei*, *Lb. curvatus*, and *Lb. paracasei* supports gut microbial diversification and homeostasis, potentially leading to more sustainable pig farming due to improved FCR.

## 1 Introduction

Considerable scientific attention has been directed toward the development and assessment of nutritional strategies aimed at maintaining health status and improving production efficiency in pigs. One such strategy is the application of probiotics as a promising multifunctional approach that can improve animal health and productivity parameters (1, 2).

Commercial probiotics used in pig nutrition have been widely reviewed, and following conclusions have been reported. One of these indicates that relatively high doses (400–600 g/ton of feed for grower–finisher pigs) are required to achieve an effective impact when enriching feed with probiotics such as BioPlus 2B, which consists of *Bacillus licheniformis* and *B. subtilis* spores (3). In addition, several limitations (e.g., stability, viability, safety, mode of action, and strain-specific effectiveness) associated with the use of probiotics in animal nutrition have been identified (4). It has also been reported that probiotic viability does not always meet expected levels (5). One potential approach to addressing this issue is the application of fed-batch fermentation (6–8). This approach may result in a synergistic effect between probiotics and postbiotics (non-viable bacterial products or metabolic byproducts produced by probiotic microorganisms that exert biological activity in the host), which has been reported to be more effective (9). In this context, fermented feed has gained interest as a promising nutritional strategy.

The effects of fermented feed are also not unequivocal. It has been reported that fermented feed may have a positive effect on average daily gain and the gain-to-feed ratio, while having no significant effect on average feed intake (10). These changes are associated with improvements in feed nutritional value, enhanced nutrient digestibility, and a reduction in antinutritional factors in feed (11–14).

Feed fermentation can be carried out using different technological approaches, namely fermentation in the liquid phase or solid-state fermentation (13, 15). Solid-state fermentation can be conditionally classified according to its application purposes. For example, when supplemented with medicinal plants, it may contribute to improved animal immunity (16–18). Alternatively, it can be applied to reduce antinutritional factors (i.e., glucosinolates, erucic acid, tannins, phytates, etc.), thereby improving feed efficiency (19–21).

The positive effects of fermented feed are associated with its ability to alter the gastrointestinal microbiota and intestinal environment in ways that enhance animal health and performance (22–24).

Our previous studies showed that LAB possess antimicrobial properties (25) and contribute to gut microbial balance by improving intestinal health in piglets (23, 24). Specifically, we demonstrated that these LAB strains (*Lb. plantarum*, *Lb. casei*, *Lb. curvatus*, and *Lb. paracasei*) inhibit the growth of several pathogenic bacteria, including *Klebsiella pneumoniae*, *Salmonella enterica*, *Pseudomonas aeruginosa*, *Acinetobacter baumannii*, *Proteus mirabilis*, methicillin-resistant *Staphylococcus aureus* (MRSA), *Enterococcus faecalis*, *Enterococcus faecium*, *Bacillus cereus*, *Streptococcus mutans*, *Enterobacter cloacae*, *Citrobacter freundii*, *Staphylococcus epidermidis*, *Staphylococcus haemolyticus*, and *Pasteurella multocida* (25). All *Lactobacillus* species are capable of lowering pH through the production of lactic acid as the end product of carbohydrate fermentation (26). A decrease in pH is caused by the production of short-chain fatty acids (SCFAs), which are generated during microbial fermentation and release hydrogen ions into the environment, making it more acidic (27). In addition, some strains synthesize antimicrobial peptides and other metabolites that inhibit the growth of pathogenic bacteria (28–30).

The hypothesis of our experiment is that the use of lactic acid bacteria (LAB) with antimicrobial properties (*Lb. plantarum*, *Lb. casei*, *Lb. curvatus*, and *Lb. paracasei*) for the production of with *Lactobacillus* spp.–fermented feed material (Lb.-FM) could be a suitable approach for improving the health status and productivity parameters of piglets.

Furthermore, to assess the effects of Lb.-FM, the profile of volatile organic compounds (VOC) in feces was analyzed. In the livestock sector, continuous efforts are being made to develop innovative tools for monitoring animal health, and various biomarkers may contribute to the advancement of precision livestock farming, with the aim of increasing productivity and improving animal health and welfare (31). In this context, the analysis of VOC may provide novel insights into nutrient metabolism and its relationships with other, more conventional indicators, such as zootechnical performance and animal health parameters.

In this study, the effect of supplementing the diet of Yorkshire/Norwegian Landrace/Duroc (J/NL/D) F2 crossbred fattening piglets with Lb.-FM was investigated. Growth performance and health parameters were evaluated, including body weight (BW), feed conversion ratio (FCR), plasma concentrations of immunoglobulins (IgA, IgM, and IgG), and the activities of aspartate aminotransferase (AST) and alanine aminotransferase (ALT). Additionally, faecal parameters were analysed, including pH, dry matter content, texture hardness, LAB, total enterobacteria counts (TEC), yeast/mold (Y/M) counts, and faecal microbial metataxonomic profiles. Furthermore, faecal VOC profiles were analysed as possible sensitive chemical markers of nutrient metabolism.

## 2 Materials and methods

### 2.1 Fermented feed material preparation

**Figure 1:**
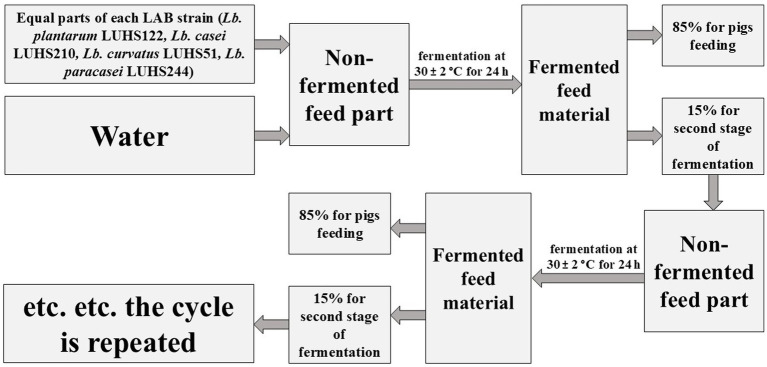
The principal scheme of the fermented feed material preparation (LAB – lactic acid bacteria).

The experimental design for feed fermentation is presented in Figure 1.

The *Lb. plantarum* LUHS122*, Lb. casei* LUHS210*, Lb. curvatus* LUHS51, and *Lb. paracasei* LUHS244 strains were obtained from the Lithuanian University of Health Sciences collection (Kaunas, Lithuania). Earlier findings from our research indicate that the listed strains are effective in limiting the growth of pathogenic and opportunistic microorganisms (25). The LAB strains were cryopreserved at −80 °C using a Microbank system (Pro-Lab Diagnostics, UK) and reactivated by separate cultivation in de Man–Rogosa–Sharpe (MRS) broth (CM0359, Oxoid Ltd., Hampshire, UK) at 30 ± 3 °C for 48 h prior to application in feed fermentation. Afterward, equal parts of each strain by volume (3% of the feed’s dry matter, v/m), containing 8.9 log₁₀ CFU/mL, were added to the feed and fermented at 30 ± 2 °C for 24 h. The composition of the fermented feed was as follows: crude protein (20.08%), crude fiber (7.34%), crude oil and fats (6.23%), lysine (1.01%), methionine (0.40%), tryptophan (0.28%), threonine (0.87%), Ca (0.33%), total P (0.64%), Na (0.02%), NaCl (0.11%), Mg (0.27%), K (0.76%), S (0.20%), starch (31.95%), and sugars (5.90%). The final moisture content of the feed was 60 g/100 g, determined by drying samples at 103 ± 2 °C to constant weight. The total mass of fermented feed was divided into two fractions: 85% was used for piglet feeding, and 15% was retained as a starter for subsequent fermentation cycles. To ensure batch-to-batch consistency, feeding was conducted using a WEDA system (Dammann & Westerkamp GmbH, Germany) equipped with integrated industrial pH and temperature sensors, which trigger alarms if predefined limits are exceeded.

### 2.2 Animals’ housing and experimental design

All animal procedures were conducted according to the EU Directive (32) of the European Parliament and of Council from 22 September 2010 on the protection of animals used for scientific purposes and Requirements for the Keeping, Maintenance and Use of Animals Intended for Science and Education Purposes, approved by the order of the Lithuanian Service (33).

Piglets (Yorkshire/Norwegian Landrace/Duroc (J/NL/D) F2 crossbred) were obtained from the “Vyturys” Agricultural Company farm (Silale district, Misuciu village, Lithuania) and, after weaning at 21 days of age, weighing an average of 6.3 kg, were transported to the “Kontvainiai” Agricultural Company farm (Klaipeda district, Kantvainiai village, Lithuania) for further fattening. The experiment began with 21-day-old piglets and lasted 40 days (till 61st day of piglets life). The animals were divided into two groups: 175 piglets in the control (C) group, which received a complete commercial feed, and 177 piglets in the Lb.-FM group, which received Lb.-FM with *Lb. plantarum*, *Lb. casei*, *Lb. curvatus*, and *Lb. paracasei*. The study was conducted at “Kontvainiai” Agricultural Company farm (Klaipeda district, Kantvainiai village, Lithuania) and at the Institute of Animal Rearing Technologies, Lithuanian University of Health Sciences (Kaunas, Lithuania). The experiment lasted 40 days.

Piglets (postweaning days 21–61) were fed age- and body weight–appropriate complete compound diets for weaned pigs. The first diet was provided from weaning until approximately 15 kg body weight or 49 days of age, after which a second diet was administered until day 61 (approximately 23 kg body weight). The dietary transition lasted 5 days, during which both diets were gradually mixed.

Piglets were housed in a barn with two climatic zones. The first zone consisted of a heated concrete area covered by an adjustable-height roof, maintaining a constant temperature of 32 °C. As the piglets aged, the roof height was gradually increased. The second zone comprised the remaining common area, maintained at 25 °C at weaning, with environmental conditions adjusted according to animal growth. Pens consisted of 30% heated concrete flooring (under the roofed area) and 70% plastic slatted flooring, where feeding and watering were provided.

A total of 24 pens (12 per side of a central corridor) were located within the barn. The feeding system enabled delivery of two different diets within the same facility via a single feeding line: fermented feed was supplied to pens on the left side and commercial feed to pens on the right side. Typically, two pens shared one feeding trough, housing approximately 44 piglets per pen. For the present study, two troughs per pen were used to ensure accurate feed intake measurements. Piglets remained in their original pens throughout the experiment and were neither mixed nor relocated.

**Figure 2:**
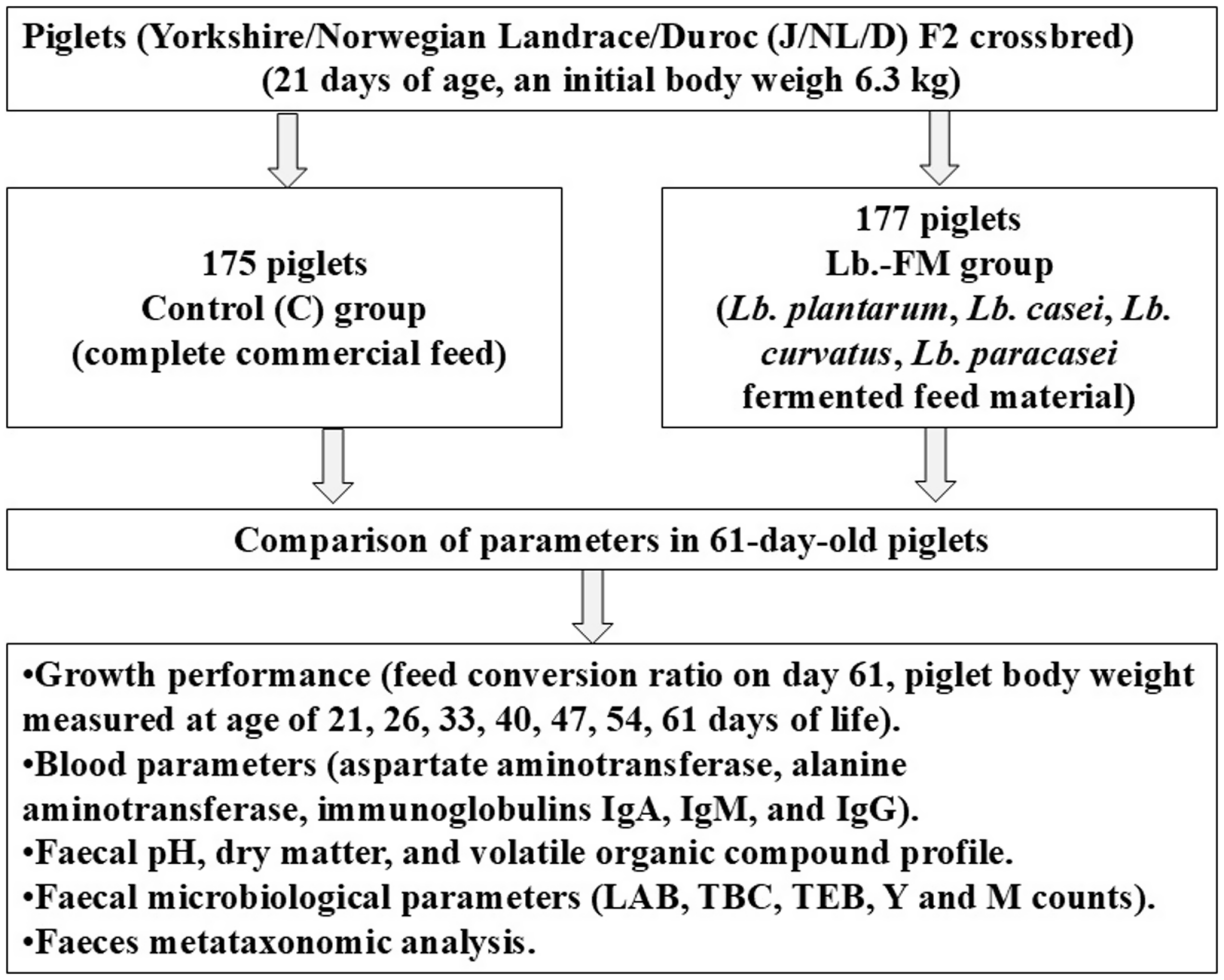
The principal scheme of the feeding experiment (Lb.-FM group – fed with *Lb. plantarum*, *Lb. casei*, *Lb. curvatus, Lb. paracasei* fermented feed material; IgA – immunoglobulin A; IgM – immunoglobulin M; IgG – immunoglobulin G; LAB – lactic acid bacteria; TBC – total bacteria count; TEC – total enterobacteria count; Y and M – yeast and mold).

Water and liquid compound feed were provided ad libitum. No antimicrobial treatments were administered. Two dietary treatments were evaluated: (i) a non-fermented basal diet and (ii) a fermented diet prepared with a combination of *Lb. plantarum* LUHS122, *Lb. casei* LUHS210, *Lb. curvatus* LUHS51, and *Lb. paracasei* LUHS244. The principal scheme of the whole experiment is shown in Figure 2.

The fermented component constituted 450 g/kg of the total diet. The basal diet was formulated in accordance with the *Nutrient Requirements of Swine* (34). Feed composition and nutritional values are provided in Table 1. Fermented feed composition is presented in Table 2. Dietary components were selected following AOAC recommendations (35).

**Table 1:** Diet composition.

**Ingredients (%)**	Control group 6–15 kg KOM-50*	**Control group** **15–24 kg** KOM-51*	**Treated group** **6–15 kg** PRO-1*	**Treated group 15-24 kg** PRO-2*
24 h fermented wheat			22.5	22.5
24 h fermented rapeseed meal			22.5	22.5
Barley	20.0	44.70	15.00	24.13
Wheat	22.66	21.00	11.10	17.49
Hulled soybean meal		19.00		
Maize	15.00			
Soybean meal	2.00		2.20	5.35
Potato protein	5.00		2.00	
Extruded rapeseed mix		5.00		
Sweet whey powder	8.00			
Sunflower oil		2.00		
Barley malt sprouts		3.00		
Corn gluten	3.00			
Nordic Soya concentrate	5.90		1.22	
Sugar beet pomace		2.00		
Sugar beet pulp	4.00		2.00	
Dextrose Monohydrate	6.50		2.00	1.00
Vegetable oil	2.90		3.65	3.47
Premix, AA and other additives, %	5.04	3.30	15.83	3.56
Nutritional value				
ME swine (MJ/kg)	14.40	13.59	13.95	13.30
Crude protein	18.00	18.86	18.50	17.5
Digestible protein	16.92	16.11	16.47	15.47
Crude fat	5.29	4.63	5.51	5.79
Crude fibre	2.81	3.85	5.03	5.15
Crude ash	5.13	4.86	5.38	5.35
Lysine	1.42	1.19	1.48	1.41
Methionine	0.55	0.51	0.55	0.53
Threonine	0.95	0.81	1.00	0.95
Tryptophan	0.30	0.25	0.30	0.29
(Ca)–Calcium	0.85	0.69	0.80	0.75
(P)–Phosphorus (total)	0.51	0.42	0.66	0.63
(Na)–Sodium	0.22	0.15	0.16	0.18
NaCl	0.89	0.50	0.77	0.82
(Mg)–Magnesium	0.12	0.10	0.18	0.19
(K)–Potassium	0.64	0.57	0.57	0.63
(S)–Sulphur	0.18	0.16	0.17	0.17
Starch	34.10	40.16	38.23	39.02
Sugar	14.31	3.77	5.84	5.12

* KOM-50, KOM-51, PRO-1, PRO-2 are serial numbers of the feed production company AB “Kauno Grūdai” (Kaunas, Lithuania). AA – amino acids.

**Table 2:** Fermented feed material composition.

Ingredients, %	Treated group 6−24 kg
24 h Fermented wheat	22.5
24 h Fermented rapeseed meal	22.5
Nutritional value	
ME (metabolic energy) swine (MJ/kg)	12.94
Crude protein	20.08
Digestible protein	18.20
Crude fat	6.23
Crude fiber	7.34
Crude ash	4.65
Lysine	1.01
Methionine	0.40
Threonine	0.87
Tryptophan	0.28
(Ca)–Calcium	0.33
(P)–Phosphorus (total)	0.64
(Na)–Sodium	0.02
NaCl	0.11
(Mg)–Magnesium	0.27
(K)–Potassium	0.78
(S)–Sulfur	0.20
Starch	31.95
Sugar	5.90

### 2.3 Evaluation of piglets’ growth performance

Piglets in both the experimental and control groups were weighed on postweaning days 21, 26, 33, 40, 47, 54, and 61 using an electronic scale (IT1000, SysTec GmbH, Bergheim, Germany). The farm infrastructure is designed to minimize animal stress during routine weighing procedures. The passage leading to the scale was constructed from materials identical to those used in the pens, including the same concrete, plastic components, and slatted flooring. The scale platform was also fitted with plastic grating identical to that in the pens, thereby reducing exposure to unfamiliar surfaces and facilitating calm animal movement. For each weighing event, the entire pen group (approximately 44 piglets) was assessed.

Feed conversion ratio (FCR) was calculated based on feed intake (87% dry matter) and body weight gain recorded on the corresponding measurement days. Feed intake data were obtained using an automated WEDA feeding system (Dammann & Westerkamp GmbH, Germany). Diets were prepared in a mixer and distributed according to a predefined feeding curve. A flow meter integrated into the system quantified the amount of feed delivered to each trough during feeding events.

### 2.4 Blood plasma analysis

For blood plasma analysis, piglets with comparable average body condition were selected from both the experimental and control groups and individually identified using ear tags. Six piglets per group were included. Blood samples were collected on postweaning days 28 and 60 prior to the morning feeding. During sampling, piglets were restrained using a snare, and blood was drawn from the jugular vein into vacuum tubes (BD Vacutainer, Plymouth, UK) using 18G × 1½″ needles. Plasma concentrations of immunoglobulins (IgA, IgM, and IgG) and the activities of aspartate aminotransferase (AST) and alanine aminotransferase (ALT) were determined using an automated biochemical analyzer in the accredited laboratory “Anteja” (Klaipeda, Lithuania). All blood sampling procedures were performed by a licensed veterinarian (Šarūnas Badaras, Veterinary practice license No. 1610) employed at the farm.

### 2.5 Evaluation of fecal parameters

#### 2.5.1 Evaluation of fecal pH, dry matter, and texture hardness

For the assessment of fecal pH, dry matter content, texture hardness, and microbiological parameters, individual fecal samples were collected from six piglets per group at baseline (day 21) and at the end of the trial (day 61). Samples were placed in sterile vials containing transport medium (Faecal Enteric Plus, Oxoid, Basingstoke, UK), stored at 4 °C, and processed on the day of collection.

#### 2.5.2 Microbiological analysis of fecal samples

To determine bacteria counts, 10 g of fecal sample were homogenized in 90 mL of sterile saline (9 g/L NaCl). Serial tenfold dilutions (10^–^^4^ to 10^–^^8^) were prepared in saline. For LAB counts determination, samples were surface-plated onto De Man, Rogosa and Sharpe (MRS) agar (CM0361, Oxoid) (36); for total enterobacteria counts (TEC) Violet Red Bile Glucose (VRBG) agar (Oxoid Ltd., Basingstoke, UK) was used (37); for Total Bacteria Count (TBC) determination plate count agar (Biolife, Milan, Italy) was used (38); for yeast/mold (Y/M) counts determination Dichloran Rose Bengal Chloramphenicol (DRBC) agar (Liofilchem, Milan, Italy) was used (39). Results were expressed as log₁₀ colony-forming units per gram of sample (log₁₀ CFU/g).

### 2.6 Metataxonomic analysis of the bacterial composition in the fecal samples of piglets’

On day 21 and on day 61 of age feces from all piglets in all the groups were collected using sterile cotton swabs. By mixing equal amounts (0.5–214 g) of individually collected feces, samples from both groups were obtained for targeted 16S rRNA sequencing. Pooled samples were stored in DNA/RNA Shield (1:10 dilution; R1100-250, Zymo Research, USA) at −70 °C until DNA extraction. DNA extraction, preparation of libraries, 16S sequencing, taxonomy assignment and interpretation of the results was performed as described previously (40).

### 2.7 Analysis of the fecal volatile compound profile

Feces were prepared for gas chromatography (GC) analysis by using solid-phase microextraction (SPME). An SPME device with Stableflex (TM) fibre, coated with a 50-μm DVB-PDMS-Carboxen™ layer (Supelco, USA), was used for sample preparation. For gas chromatography–mass spectrometry (GC–MS), a GCMS-QP2010 (Shimadzu, Japan) was used. The gas chromatograph was equipped with an AOC-5000 Plus Shimadzu autosampler, upgraded with an SPME analysis kit. Analysis was performed according to the procedure described by Vadopalas et al. (41).

### 2.8 Statistical analysis

Normality of data distribution was assessed using the Shapiro–Wilk test. In order to compare the differences in parameter means between the different groups (C and Lb.-FM), an independent samples *t*-test was used (for growth performance *n* = 177 from each group); for blood plasma parameters: at the beginning and at the end of experiment, *n* = 6 from each group; for the pH, dry matter, texture, VC profile, and microbiological parameters of fecal samples before (on day 21) and after (at day 61) the experiment, *n* = 6 from each group; for metataxonomic analysis of the bacterial composition in the fecal samples, at the beginning of experiment, samples were collected from 12 piglets from each group and a single pooled sample was prepared for microbiome profiling, after the experiment, feces from 12 piglets from the C and FFM groups were collected and thereafter, two pooled samples, representing each of the groups, were prepared and analyzed. The *p-*values of factor (diet) influence were determined by multivariate tests of between-subjects effects. Baseline measurements were used as covariates to account for the experimental conditions. The mean values were compared using Duncan’s multiple range *post hoc* test with the significance level defined at *p* ≤ 0.05. In the tables, the results are presented as mean values with pooled standard errors. Also, Pearson’s correlations between characteristics were calculated, strength of the correlations was interpreted according to Evans (42). Correlations were considered significant when *p ≤* 0.05. For the quality control of taxonomical identification, standard (D6300, Zymo Research, Murphy Ave. Irvine, CA, USA) of mixed known bacterial cultures were sequenced together with sample sequencing. The number of reads of each genera/species from the total number of reads in samples was counted. Relative abundance of the genera/species in samples then were compared between the groups.

## 3 Results

### 3.1 Piglets’ body weight and feed conversion ratio

**Figure 3:**
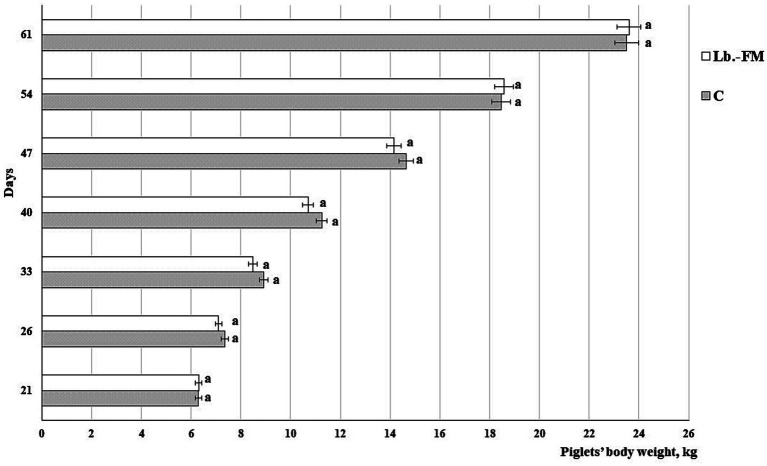
Piglets’ body weight, kg (C – control group, received a complete commercial feed; Lb.-FM group, received fermented feed material with *Lb. plantarum*, *Lb. casei*, *Lb.* curvatus, and *Lb. paracasei*); ^a^ letter indicates no significant differences (*p* ≤ 0.05). The data are presented as the mean ± standard deviation (*n* = 175/C group, *n* = 177/C Lb.-FM group).

**Figure 4:**
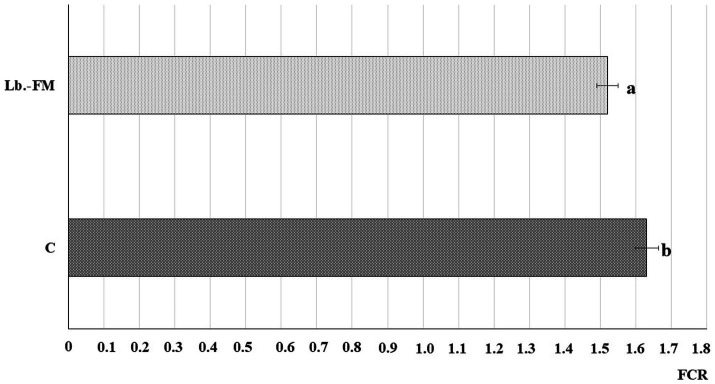
Feed conversion ratio on day 61st (FCR – feed conversion ratio; C – control group, received a complete commercial feed; Lb.-FM group, received fermented feed material with *Lb. plantarum*, *Lb. casei*, *Lb. curvatus*, and *Lb. paracasei*; ^a, b^ different letters indicate significant differences, *p* ≤ 0.05, *n* = 175/C group, *n* = 177/C Lb.-FM group).

Despite the absence of significant differences in piglet body weight between the control group, which received a complete commercial feed, and the Lb.-FM group, which received fermented feed material containing *Lb. plantarum*, *Lb. casei*, *Lb. curvatus*, and *Lb. paracasei* (Figure 3), the feed conversion ratio (FCR) was lower in the Lb.-FM group compared with the control group (Figure 4).

### 3.2 Piglets’ blood plasma parameters

The blood plasma parameters of piglets at 21 and 61 days of age are presented in Table 3. When analyzing time-related differences, higher concentrations of IgM and IgG were observed in the blood plasma of 61-day-old piglets compared with 21-day-old piglets in both groups (control and Lb.-FM). In the control group, IgM concentration was on average 2.07 times higher, while IgG concentration was 1.53 times higher at 61 days of age. In the Lb.-FM group, IgM concentration was 2.43 times higher and IgG concentration was 2.01 times higher compared with the values observed at 21 days of age. Furthermore, the ALT concentration increased at day 61 compared with the plasma samples of 21-day-old piglets. In the control group, ALT levels were on average 1.76 times higher, while in the Lb.-FM group they were 1.86 times higher.

**Table 3:** Blood plasma parameters of 21 and 61-day-old piglets.

Blood parameters		Treatments		*p*	
		C	Lb.-FM	C × Lb.-FM	Significance of the analyzed factor (diet)
Immunoglobulin A (IgA), g/L	21	<0.330	<0.330	–	–
	61	<0.330	<0.330	–	–
Immunoglobulin M (IgM), g/L	21	0.247 ± 0.083^a^	0.255 ± 0.115^a^	0.786	0.806
	61	0.513 ± 0.257^b^	0.620 ± 0.300^b^	0.311	0.283
Immunoglobulin G (IgG), g/L	21	2.12 ± 0.772^a^	2.03 ± 1.22^a^	0.581	0.507
	61	3.25 ± 0.882^b^	4.07 ± 3.35^b^	0.378	0.313
ALT, U/L	21	57.2 ± 21.8^a^	56.7 ± 16.3^a^	0.948	0.940
	61	100.5 ± 48.5^b^	105.7 ± 67.3^b^	0.838	0.802
AST, U/L	21	48.3 ± 18.7^a^	40.5 ± 14.5^a^	0.173	0.170
	61	45.3 ± 13.7^a^	45.3 ± 18.3^b^	1.000	1.000

C – control group – fed with basal non-fermented diet; Lb.-FM group – fed with *Lb. plantarum*, *Lb. casei*, *Lb. curvatus, Lb. paracasei* fermented feed material; 21th – at the beginning of the experiment; 61th – at the end of the experiment; ALT – alanine aminotransferase; AST – aspartate aminotransferase. ^a, b^ Different letters indicate significant time-related differences (*p* ≤ 0.05). The data are presented as the mean ± standard deviation (*n* = 6/group).

A slightly higher AST concentration (on average 11.9%) was detected in the Lb.-FM group at 61 days of age compared with 21 days.

When comparing the control and Lb.-FM groups, no significant differences in blood plasma parameters were detected at either 21 or 61 day of age. Overall, diet did not have a significant effect on the analyzed blood parameters.

### 3.3 Piglets’ fecal pH, dry matter, and texture hardness

The pH, dry matter, and texture hardness of piglets’ feces are shown in Table 4. At 61 days of age, fecal texture hardness was higher in the control group, being on average 2.27 times greater than in the Lb.-FM group. However, no significant differences between the control and Lb.-FM groups were observed in faecal pH (at 21 and 61 days), dry matter content (at 21 and 61 days), or faecal texture hardness at 21 days of age.

**Table 4:** The pH, dry matter and texture hardness of piglet feces.

**Fecal parameters**		**Treatments**		***p***	
		C	Lb.-FM	C × Lb.-FM	Significance of the analyzed factor (diet)
pH	21	6.36 ± 0.17^a^	6.12 ± 0.08^a^	0.504	0.480
	61	6.25 ± 0.14^a^	6.02 ± 0.08^a^	0.744	0.665
Dry matter (%)	21	32.42 ± 1.95^a^	42.06 ± 1.54^a^	0.980	0.979
	61	30.91 ± 1.89^a^	39.11 ± 1.70^a^	0.657	0.601
Texture hardness (mJ)	21	0.60 ± 0.06^a^	0.34 ± 0.03^a^	0.111	0.267
	61	0.59 ± 0.05^a^	0.26 ± 0.02^a^	0.050	0.087

C – control group – fed with basal non-fermented diet; Lb.-FM group – fed with *Lb. plantarum*, *Lb. casei*, *Lb. curvatus, Lb. paracasei* fermented feed material; 21th–at the beginning of the experiment; 61th–at the end of the experiment. ^a^ Letters indicate no significant time-related differences (*p* ≤ 0.05). The data are presented as the mean ± standard deviation (*n* = 6/group).

### 3.4 Microbiological parameters of piglets’ feces

Microbiological parameters of piglet fecal samples are shown in Table 5. No significant differences were observed in the microbiological parameters of piglet fecal samples between the groups. Diet also had no significant effect on these parameters, except for time-related differences in fecal total bacterial count (TBC) in the control group.

**Table 5:** Microbiological parameters of piglets’ fecal samples.

**Fecal parameters**		**Treatments**		***p***	
		C	Lb.-FM	C × Lb.-FM	Significance of the analyzed factor (diet)
LAB	21	4.85 ± 0.24^a^	6.50 ± 0.23^a^	0.183	0.098
	61	5.05 ± 0.29^a^	7.57 ± 0.27^a^	0.947	0.937
TBC	21	6.12 ± 0.14^b^	8.02 ± 0.19^a^	0.197	0.147
	61	5.63 ± 0.15^a^	7.41 ± 0.12^a^	0.139	0.106
TEC	21	6.15 ± 0.17^a^	5.85 ± 0.16^a^	0.413	0.311
	61	5.13 ± 0.09^a^	5.61 ± 0.19^a^	0.397	0.293
M and Y	21	4.99 ± 0.23^a^	5.84 ± 0.20^a^	0.506	0.430
	61	4.65 ± 0.19^a^	4.82 ± 0.24^a^	0.799	0.759

C – control group – fed with basal non-fermented diet; *Lb.-FM* group – fed with *Lb. plantarum*, *Lb. casei*, *Lb. curvatus*, *Lb. paracasei* fermented feed material; 21th–at the beginning of the experiment; 61th–at the end of the experiment; LAB–lactic acid bacteria; TBC–total bacteria count; TEC–total enterobacteria count; Y and M–yeast and mold. ^a, b^ Different letters indicate significant time-related differences (*p* ≤ 0.05). The data are presented as the mean ± standard deviation (*n* = 6/group).

### 3.5 Metataxonomic composition of fecal microbiota

**Figure 5:**
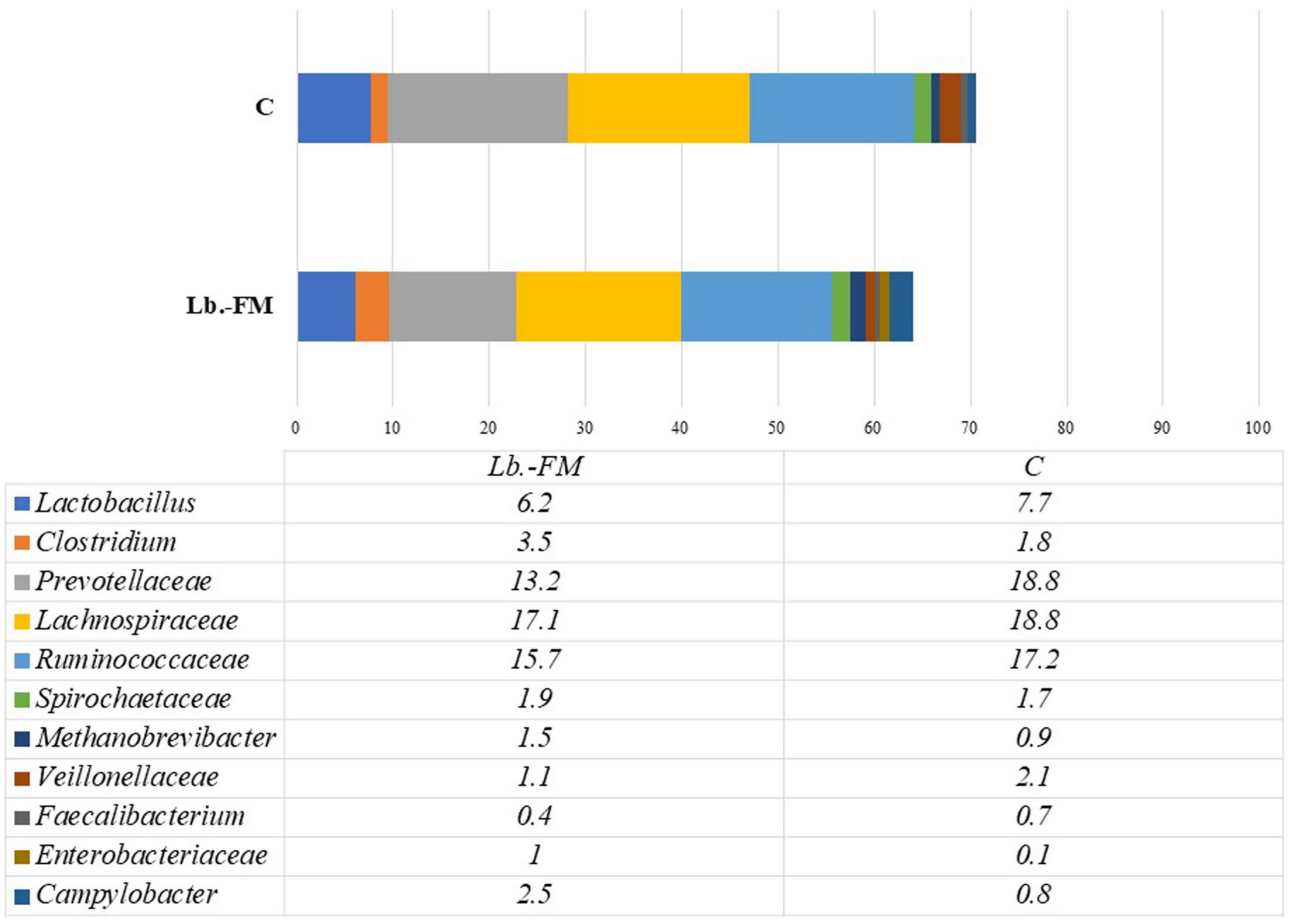
Descriptive comparison of the most prevalent bacteria in pooled fecal samples of pigs at the beginning of the experiment, day 21.

The most prevalent bacterial taxa at the beginning of the experiment in piglets’ feces (day 21) are presented in Figure 5. The main bacteria were anaerobes from families Ruminococcaceae, Lachnospiraceae and Prevotellaceae which prevalence were from 46 to 54.8% from all bacterial amount in Lb.-FM and C groups, respectively. *Lactobacillus* in both groups also was prevalent (6.2–7.7%) without obvious difference between the groups. The rest mostly prevalent bacteria included Spirochetaceae, Veillonellaceae, Enterobacteriaceae, *Clostridium*, *Campylobacter*, and *Methanobrevibacter*.

**Figure 6:**
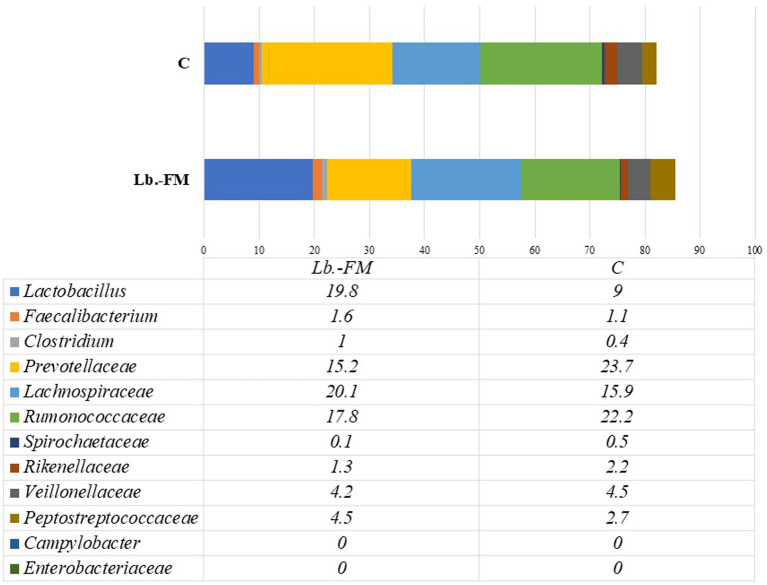
Descriptive comparison of the most prevalent bacteria in pooled fecal samples of pigs at the end of the experiment, day 61.

Microbial profiles in pig feces at the end of the experiment is presented in Figure 6.

At the end of the experiment, on day 61, microbial composition slightly changed in both animal groups however, Ruminococcaceae, Lachnospiraceae and Prevotellaceae remained the most prevalent families, with slightly increased numbers, and accounted for 53.1 and 61.8% from all bacterial count in Lb.-FM and C groups, respectively. The main difference between the groups at the end of experiment was in the amount of *Lactobacillus*, which in Lb.-FM was the second most prevalent genus (19.8%) with 2.2 times higher percental numbers than in the control group. Although the species of the vast majority lactobacilli were unidentified up to the species level, identified *Lactobacillus* included *L. pontis*, *L. kitasatonis*, *L. amylovorus, L. mucosae* and *L. panis*. Experimental group also has higher numbers of Peptostreptococcaceae (with the dominant genera *Romboutsia* and *Terrisporobacter*) and *Faecalibacterium.* Control group contained higher amount of Prevotellaceae and Ruminococcaceae as well as Spirochaetaceae, Veilonellaceae and Rikenellaceae. *Campylobacter* and *Enterobacteriaceae* on day 61 significantly decreased in faeces of both groups (the total prevalence <0.01%).

### 3.6 Volatile organic compound profiles of piglets’ feces

The VOC profiles of piglet faeces are presented in Tables 6, 8, 10. Correlations and their significance between individual VOCs and faecal microbiological parameters, pH, dry matter content, and texture hardness are presented in Tables 7, 9, 11, respectively.

**Table 6:** Volatile organic compound (carboxylic acids, fatty acid methyl esters, fatty acids, indole derivatives, and cyclic ethers, % from the total volatile compound) profiles of piglets’ faeces.

Group of the volatile organic compound	Volatile organic compound	Days	C	Lb.-FM	C × Lb.-FM	Significance of the analyzed factor (diet)
Carboxylic acids: aliphatic (saturated) acids	Acetic acid	21	2.26 ± 0.62^a^	2.23 ± 0.52^a^	0.925	0.929
		61	1.66 ± 0.25^a^	1.84 ± 0.53^b^	0.495	0.465
	Propionic acid	21	4.04 ± 1.12^a^	2.37 ± 1.07^a^	0.887	0.882
		61	3.96 ± 0.85^a^	2.51 ± 1.17^b^	0.773	0.838
	Butanoic acid	21	22.2 ± 8.56^a^	24.9 ± 8.11^a^	0.684	0.583
		61	18.0 ± 7.69^a^	17.4 ± 7.78^a^	0.910	0.904
	3-methylbutanoic acid	21	1.65 ± 0.56^a^	1.88 ± 0.40^a^	0.378	0.424
		61	2.41 ± 1.02^a^	2.17 ± 0.92^a^	0.606	0.672
	2-methylbutyric acid	21	3.93 ± 3.61^a^	6.37 ± 3.96^a^	0.118	0.291
		61	17.3 ± 7.36^b^	15.5 ± 5.33^b^	0.673	0.644
	Pentanoic acid	21	30.5 ± 7.90^a^	31.4 ± 5.45^b^	0.792	0.830
		61	21.0 ± 3.39^a^	21.4 ± 3.99^a^	0.880	0.868
	4-Methylvaleric acid	21	0.228 ± 0.231	0.506 ± 0.298	0.172	0.101
		61	nd	nd	–	–
	Hexanoic acid	21	4.18 ± 4.84	2.75 ± 2.67	0.483	0.529
		61	nd	nd	–	–
	Heptanoic acid	21	0.484 ± 1.19^a^	0.307 ± 0.476^a^	0.670	0.742
		61	0.075 ± 0.183^a^	0.877 ± 1.27^a^	0.204	0.158
	Octanoic acid	21	0.097 ± 0.033^a^	0.086 ± 0.050^a^	0.418	0.657
		61	0.063 ± 0.036^a^	0.067 ± 0.030^a^	0.853	0.829
	Nonanoic acid	21	0.037 ± 0.007^a^	0.051 ± 0.024^a^	0.156	0.196
		61	0.075 ± 0.023^b^	0.078 ± 0.027^a^	0.823	0.802
Carboxylic acids: aromatic acids	Benzeneacetic acid	21	0.170 ± 0.148^a^	0.229 ± 0.173^a^	0.428	0.538
		61	0.898 ± 0.336^b^	0.808 ± 0.180^b^	0.630	0.573
	Hydrocinnamic acid	21	0.739 ± 0.192^a^	0.901 ± 0.211^a^	0.326	0.196
		61	0.729 ± 0.274^a^	0.661 ± 0.241^a^	0.701	0.658
Carboxylic acid (branched fatty acid)	Isohexanoic acid	21	nd	nd	–	–
		61	0.550 ± 0.298	0.386 ± 0.232	0.249	0.311
Fatty acid methyl ester	Hexadecanoic acid methyl ester	21	nd	nd	–	–
		61	0.020 ± 0.014	0.032 ± 0.025	0.275	0.341
Fatty acid (saturated carboxylic acid)	Hexadecanoic acid	21	nd	–	–	–
		61	0.016 ± 0.033	0.175 ± 0.068	0.673	0.634
Indole derivatives	Indole	21	0.586 ± 0.430^a^	0.682 ± 0.561^a^	0.303	0.748
		61	0.689 ± 0.364^a^	0.736 ± 0.541^a^	0.882	0.863
	Skatole	21	7.36 ± 2.94^a^	5.02 ± 2.33^a^	0.194	0.157
		61	4.51 ± 1.58^a^	4.26 ± 2.17^a^	0.814	0.829
Cyclic ether	Hexadecene epoxide	21	nd	nd	–	–
		61	0.379 ± 0.167	0.406 ± 0.159	0.741	0.783

C–control group–fed with basal non-fermented diet; *Lb.-FM* group – fed with *Lb. plantarum*, *Lb. casei*, *Lb. curvatus*, *Lb. paracasei* fermented feed material; 21th – at the beginning of the experiment; 61th – at the end of the experiment. ^a, b^ Different letters indicate significant time-related differences (*p* ≤ 0.05). The data are presented as the mean ± standard deviation (*n* = 6/group).

**Table 7:** Correlations and their significance between individual volatile organic compounds (carboxylic acids, fatty acid methyl esters, fatty acids, indole derivatives, and cyclic ethers) in piglet faeces and faecal microbiological parameters, pH, dry matter content, and texture hardness.

**Volatile compound**	***r* and *p*-values**	**LAB**	**TBC**	**TEC**	**Y and M**	**pH**	**DM**	**Texture**
Acetic acid	*r*	−0.156	0.072	**0.530** ^**^	**0.518** ^**^	0.070	−0.017	0.024
	*p*	0.466	0.737	**0.008**	**0.010**	0.745	0.936	0.913
Propionic acid	*r*	−0.293	0.148	**0.453** ^*^	0.295	0.292	−0.001	0.259
	*p*	0.165	0.491	**0.026**	0.161	0.166	0.996	0.222
Butanoic acid	*r*	−0.148	0.160	0.133	−0.029	0.198	0.128	0.141
	*p*	0.490	0.454	0.536	0.893	0.354	0.552	0.510
3-methylbutanoic acid	*r*	0.179	−0.117	0.062	−0.038	**−0.435** ^*^	0.051	0.002
	*p*	0.402	0.588	0.774	0.859	**0.034**	0.813	0.992
2-methylbutyric acid	*r*	0.262	−0.231	−0.369	−0.167	**−0.434** ^*^	−0.077	−0.141
	*p*	0.215	0.277	0.076	0.436	**0.034**	0.719	0.510
Pentanoic acid	*r*	−0.218	0.234	0.220	0.273	0.346	0.098	0.175
	*p*	0.306	0.272	0.301	0.197	0.097	0.650	0.415
4-Methylvaleric acid	*r*	−0.006	**0.438** ^*^	0.175	0.404	0.143	0.342	−0.120
	*p*	0.979	**0.032**	0.414	0.050	0.506	0.102	0.576
Hexanoic acid	*r*	−0.159	0.119	0.306	0.082	0.197	0.116	−0.044
	*p*	0.458	0.580	0.146	0.703	0.355	0.588	0.837
Heptanoic acid	*r*	0.322	0.150	0.033	−0.298	−0.093	0.220	−0.311
	*p*	0.125	0.485	0.880	0.157	0.664	0.302	0.140
Octanoic acid	*r*	−0.059	0.157	0.061	0.179	0.198	0.003	0.069
	*p*	0.784	0.464	0.777	0.402	0.355	0.991	0.747
Nonanoic acid	*r*	0.347	0.141	−0.344	−0.045	−0.297	−0.023	−0.226
	*p*	0.097	0.512	0.099	0.834	0.159	0.916	0.288
Benzeneacetic acid	*r*	0.256	−0.255	−0.322	−0.325	−0.324	−0.179	−0.063
	*p*	0.228	0.229	0.125	0.121	0.122	0.403	0.768
Hydrocinnamic acid	*r*	−0.130	0.169	0.092	0.220	0.225	0.087	−0.172
	*p*	0.546	0.430	0.669	0.301	0.291	0.687	0.422
Isohexanoic acid	*r*	0.018	−0.0265	−0.319	−0.128	−0.075	−0.347	−0.007
	*p*	0.933	0.212	0.128	0.551	0.728	0.097	0.974
Hexadecanoic acid methyl ester	*r*	**0.423** ^*^	0.138	−0.277	−0.200	**−0.477** ^*^	0.039	−0.151
	*p*	**0.039**	0.519	0.191	0.349	**0.018**	0.858	0.481
Hexadecanoic acid	*r*	0.302	−0.132	**−0.458** ^*^	−0.269	−0.322	−0.189	−0.0163
	*p*	0.152	0.538	**0.024**	0.203	0.125	0.377	0.446
Indole	*r*	−0.006	0.122	−0.029	0.183	0.109	−0.0099	0.077
	*p*	0.980	0.571	0.892	0.391	0.611	0.647	0.722
Skatole	*r*	−0.208	−0.076	0.102	0.118	0.195	−0.031	−0.101
	*p*	0.329	0.725	0.636	0.583	0.361	0.886	0.637
Hexadecene epoxide	*r*	0.208	−0.199	−0.396	−0.248	−0.157	−0.244	−0.147
	*p*	0.329	0.352	0.056	0.243	0.465	0.251	0.494

LAB–lactic acid bacteria; TBC–total bacteria count; TEC–total enterobacteria count; Y and M–yeast and mold; DM–dry matter. *r*–Pearson Correlation; *p*–significance; ** – correlation is significant at the 0.01 level (two-tailed); * – correlation is significant at the 0.05 level (two-tailed). Correlation interpreted as significant, when *p* ≤ 0.05. Bold values indicate significant correlations (*p* ≤ 0.05).

**Table 8:** Volatile organic compound (aromatic hydrocarbons, α,β-unsaturated aldehyde, phenols, esters, alcohols, % from the total volatile compound) profiles of piglets’ feces.

**Group of the volatile organic compound**	**Volatile organic compound**	**Days**	**C**	***Lb.-FM***	**C × *Lb.-FM***	**Significance of the analysed factor (diet)**
Aromatic hydrocarbons	1,3-Di-tert-butylbenzene	21	0.217 ± 0.230	0.225 ± 0.141	0.952	0.947
		61	nd	nd	–	–
	Benzaldehyde	21	0.240 ± 0.227^a^	0.232 ± 0.222^a^	0.960	0.952
		61	0.328 ± 0.180^a^	0.214 ± 0.083^a^	0.270	0.190
α,β-Unsaturated aldehyde	Non-(2E)-enal	21	nd	nd	–	–
		61	0.093 ± 0.080	0.043 ± 0.018	0.192	0.165
Phenols	p-cresol	21	12.9 ± 4.27^a^	11.2 ± 5.20^a^	0.650	0.552
		61	23.0 ± 4.39^b^	24.5 ± 6.12^b^	0.649	0.629
	4-ethylphenol	21	0.910 ± 0.148^a^	1.19 ± 1.33^a^	0.629	0.621
		61	0.806 ± 1.06^a^	0.408 ± 0.252^a^	0.445	0.391
	2,6-Di-tert-butyl-4-methylphenol	21	0.196 ± 0.110	0.134 ± 0.052	0.211	0.241
		61	nd	nd	–	–
	Butylhydroxytoluene	21	nd	nd	–	–
		61	0.341 ± 0.124	0.082 ± 0.029	0.003	<0.001
Esters	n-Amyl isovalerate	21	0.190 ± 0.184^a^	0.320 ± 0.359^a^	0.535	0.449
		61	0.079 ± 0.115^a^	0.106 ± 0.142^a^	0.778	0.726
	Pentyl isovalerate	21	0.022 ± 0.024	0.063 ± 0.087	0.338	0.284
		61	nd	nd	–	–
	2-Ethyl-3-hydroxyhexyl 2-methylpropanoate	21	0.035 ± 0.010^b^	0.042 ± 0.026^a^	0.657	0.568
		61	0.016 ± 0.008^a^	0.019 ± 0.006^a^	0.468	0.434
	11-Dodecen-1-yl acetate	21	0.874 ± 0.348	0.649 ± 0.338	0.311	0.283
		61	nd	nd	–	–
	Butyl 2-methylbutanoate	21	nd	nd	–	–
		61	0.021 ± 0.035	0.026 ± 0.031	0.825	0.788
	3-methylbutanoic acid butyl ester	21	nd	nd	–	–
		61	0.036 ± 0.048	0.034 ± 0.044	0.973	0.967
	Benzeneacetic acid methyl ester	21	nd	nd	–	–
		61	0.005 ± 0.008	0.020 ± 0.032	0.355	0.287
	Ethyl hydrocinnamate	21	nd	nd	–	–
		61	0.024 ± 0.059^a^	0.033 ± 0.053^a^	0.815	0.784
Alcohols	3-Phenylpropanol	21	0.049 ± 0.027^a^	0.071 ± 0.038^a^	0.233	0.279
		61	nd	nd	–	–
	Pentadecanol	21	0.523 ± 0.238	0.457 ± 0.147	0.628	0.576
		61	nd	nd	–	–
	Z-2-Dodecenol	21	nd	nd	–	–
		61	0.181 ± 0.074	0.216 ± 0.098	0.568	0.498
	Cyclododecanol	21	nd	nd	–	–
		61	0.442 ± 0.256	0.509 ± 0.219	0.577	0.639

C–control group–fed with basal non-fermented diet; *Lb.-FM* group–fed with *Lb. plantarum*, *Lb. casei*, *Lb. curvatus*, *Lb. paracasei* fermented feed material; 21th–at the beginning of the experiment; 61th–at the end of the experiment. ^a, b^ Different letters indicate significant time-related differences (*p* ≤ 0.05). The data are presented as the mean ± standard deviation (*n* = 6/group).

**Table 9:** Correlations and their significance between individual volatile organic compounds (aromatic hydrocarbons, α,β-unsaturated aldehyde, phenols, esters, alcohols, % from the total volatile compound) in piglet feces and fecal microbiological parameters, pH, dry matter content, and texture hardness.

**Volatile compound**	***r* and *p* values**	**LAB**	**TBC**	**TEC**	**Y and M**	**pH**	**DM**	**Texture**
1,3-Di-tert-butylbenzene	*r*	−0.166	0.178	0.344	0.363	0.205	0.127	0.143
	*p*	0.438	0.406	0.100	0.081	0.337	0.553	0.504
Benzaldehyde	*r*	−0.092	−0.225	0.009	0.022	−0.049	−0.105	0.172
	*p*	0.668	0.290	0.966	0.919	0.820	0.627	0.422
Non-(2E)-enal	*r*	−0.177	−0.330	**−0.474** ^*^	−0.106	0.192	−0.404	−0.003
	*p*	0.409	0.115	**0.019**	0.622	0.369	0.050	0.989
p-cresol	*r*	0.283	−0.239	−0.292	−0.265	−0.329	−0.169	−0.124
	*p*	0.180	0.260	0.166	0.210	0.117	0.431	0.565
4-ethylphenol	*r*	−0.179	0.157	−0.080	0.111	0.343	−0.002	−0.053
	*p*	0.402	0.464	0.711	0.607	0.100	0.992	0.806
2,6-Di-tert-butyl-4-methylphenol	*r*	−0.324	0.054	**0.458** ^*^	0.362	0.262	0.062	0.186
	*p*	0.122	0.804	**0.024**	0.082	0.216	0.775	0.384
Butylhydroxytoluene	*r*	−0.337	**−0.546** ^**^	**−0.518** ^**^	−0.303	0.236	**−0.553** ^**^	0.242
	*p*	0.108	**0.006**	**0.010**	0.150	0.266	**0.005**	0.254
n-Amyl isovalerate	*r*	−0.020	0.270	−0.013	−0.111	0.151	0.249	−0.024
	*p*	0.927	0.202	0.951	0.605	0.481	0.240	0.910
Pentyl isovalerate	*r*	0.008	0.357	0.055	0.069	0.061	0.328	−0.154
	*p*	0.972	0.087	0.798	0.748	0.778	0.117	0.472
2-Ethyl-3-hydroxyhexyl 2-methylpropanoate	*r*	−0.010	0.385	0.213	0.212	0.126	0.269	−0.075
	*p*	0.961	0.064	0.317	0.321	0.558	0.205	0.727
11-Dodecen-1-yl acetate	*r*	−0.294	0.128	**0.463** ^*^	0.363	0.323	0.078	0.115
	*p*	0.163	0.552	**0.023**	0.081	0.124	0.716	0.593
Butyl 2-methylbutanoate	*r*	0.067	−0.047	−0.198	−0.187	0.038	−0.133	0.071
	*p*	0.754	0.826	0.354	0.381	0.858	0.536	0.743
3-methylbutanoic acid butyl ester	*r*	−0.012	−0.129	−0.250	−0.168	0.085	−0.183	0.082
	*p*	0.955	0.549	0.239	0.434	0.695	0.393	0.702
Benzeneacetic acid methyl ester	*r*	0.316	0.154	−0.229	−0.155	−0.193	0.088	−0.204
	*p*	0.132	0.472	0.282	0.470	0.366	0.683	0.338
Ethyl hydrocinnamate	*r*	0.047	−0.043	−0.218	−0.308	0.015	−0.093	0.164
	*p*	0.828	0.841	0.306	0.144	0.946	0.664	0.443
3-Phenylpropanol	*r*	−0.063	0.352	0.368	0.284	0.127	0.265	−0.001
	*p*	0.769	0.091	0.077	0.178	0.554	0.211	0.994
Pentadecanol	*r*	−0.261	0.179	**0.498** ^*^	**0.428** ^*^	0.256	0.140	0.124
	*p*	0.218	0.403	**0.013**	**0.037**	0.227	0.514	0.565
Z-2-Dodecenol	*r*	0.287	−0.218	−0.303	−0.242	−0.274	−0.178	−0.238
	*p*	0.175	0.307	0.150	0.255	0.195	0.404	0.263
Cyclododecanol	*r*	0.253	−0.170	−0.392	−0.271	−0.142	−0.198	−0.221
	*p*	0.232	0.427	0.058	0.200	0.508	0.355	0.300

LAB–lactic acid bacteria; TBC–total bacteria count; TEC–total enterobacteria count; Y and M–yeast and mold; DM–dry matter. *r*–Pearson Correlation; *p*–significance; ** – correlation is significant at the 0.01 level (two-tailed); * – correlation is significant at the 0.05 level (two-tailed). Correlation interpreted as significant, when *p* ≤ 0.05. Bold values indicate significant correlations (*p* ≤ 0.05).

**Table 10:** Volatile organic compound (aldehydes, alkanes, alkynes, alkenes, terpenes, ketones, % from the total volatile compound) profiles of piglets’ feces.

Group of the volatile organic compound	Volatile organic compound	Days	C	*Lb.-FM*	C × *Lb.-FM*	Significance of the analyzed factor (diet)
Aldehydes	Nonanal	21	0.177 ± 0.089^b^	0.161 ± 0.045^b^	0.765	0.714
		61	0.087 ± 0.046^a^	0.075 ± 0.025^a^	0.490	0.597
	Decanal	21	0.070 ± 0.024^a^	0.075 ± 0.036^b^	0.843	0.790
		61	0.041 ± 0.017^a^	0.051 ± 0.016^a^	0.255	0.325
	Dodecanal	21	0.361 ± 0.144^b^	0.353 ± 0.159^b^	0.946	0.927
		61	0.154 ± 0.070^a^	0.173 ± 0.059^a^	0.599	0.619
	Tridecanal <n->	21	0.348 ± 0.172^a^	0.270 ± 0.090^a^	0.423	0.349
		61	0.211 ± 0.102^a^	0.237 ± 0.111^a^	0.502	0.687
	Pentadecanal	21	1.02 ± 0.358^b^	0.752 ± 0.287^b^	0.279	0.191
		61	0.158 ± 0.044^a^	0.195 ± 0.088^a^	0.444	0.377
	Hexadecanal	21	1.20 ± 0.690^a^	0.877 ± 0.305^a^	0.320	0.321
		61	0.515 ± 0.279^a^	0.481 ± 0.150^a^	0.767	0.800
	cis-9-Hexadecenal	21	0.287 ± 0.151	0.225 ± 0.089	0.466	0.407
		61	nd	nd	–	–
	(2E)-2-Tetradecenal	21	nd	nd	–	–
		61	0.601 ± 0.209	0.790 ± 0.404	0.123	0.333
	Tetradecanal	21	0.980 ± 0.479^a^	0.733 ± 0.238^a^	0.337	0.284
		61	0.837 ± 0.389^a^	1.14 ± 0.727^a^	0.158	0.383
	(Z)-7-Hexadecenal	21	nd	nd	–	–
		61	0.108 ± 0.054	0.086 ± 0.064	0.589	0.545
	Octadecanal	21	nd	nd	–	–
		61	0.044 ± 0.024	0.042 ± 0.029	0.874	0.893
Alkanes	Dodecane	21	0.111 ± 0.053^b^	0.121 ± 0.050^b^	0.772	0.750
		61	0.031 ± 0.011^a^	0.031 ± 0.004^a^	0.919	0.908
	Tridecane	21	0.093 ± 0.057^a^	0.070 ± 0.031^a^	0.191	0.423
		61	0.074 ± 0.019^a^	0.072 ± 0.039^a^	0.891	0.917
	Tetradecane	21	0.104 ± 0.042^a^	0.082 ± 0.017^b^	0.211	0.268
		61	0.054 ± 0.032^a^	0.047 ± 0.007^a^	0.554	0.577
	Hexadecane	21	0.146 ± 0.089^a^	0.109 ± 0.026^a^	0.413	0.346
		61	0.089 ± 0.042^a^	0.118 ± 0.037^a^	0.311	0.235
	Heptadecane	21	0.227 ± 0.197^a^	0.143 ± 0.047^a^	0.374	0.330
		61	0.102 ± 0.046^a^	0.135 ± 0.024^a^	0.189	0.154
	Octadecane	21	0.179 ± 0.136^a^	0.126 ± 0.045^a^	0.424	0.385
		61	0.060 ± 0.009^a^	0.078 ± 0.036^a^	0.317	0.266
	Nonadecane	21	nd	nd	–	–
		61	0.045 ± 0.064	0.082 ± 0.132	0.503	0.551
	Eicosane	21	nd	nd	–	–
		61	0.184 ± 0.130	0.225 ± 0.234	0.668	0.713
Alkynes	1-Octadecyne	21	nd	nd	-	-
		61	0.060 ± 0.034	0.071 ± 0.028	0.570	0.549
Alkene	1-Nonadecene	21	nd	nd	–	–
		61	0.001 ± 0.003	nd	–	–
Terpene	β-Gurjunene	21	0.083 ± 0.087^a^	0.052 ± 0.010^a^	0.383	0.416
		61	0.051 ± 0.015^a^	0.041 ± 0.022^a^	0.252	0.402
Ketones	Methyl octyl ketone	21	nd	nd	–	–
		61	0.032 ± 0.012	0.037 ± 0.029	0.583	0.674
	Heptyl ketone	21	nd	nd	–	–
		61	0.046 ± 0.025	0.047 ± 0.026	0.906	0.920
	Decyl methyl ketone	21	nd	nd	–	–
		61	0.075 ± 0.019	0.065 ± 0.018	0.355	0.393
	Geranyl acetone	21	nd	nd	–	–
		61	0.025 ± 0.005	0.020 ± 0.008	0.271	0.222

C–control group – fed with basal non-fermented diet; *Lb.-FM* group–fed with *Lb. plantarum*, *Lb. casei*, *Lb. curvatus*, *Lb. paracasei* fermented feed material; 21th–at the beginning of the experiment; 61th–at the end of the experiment. ^a, b^ Different letters indicate significant time-related differences (*p* ≤ 0.05). The data are presented as the mean ± standard deviation (*n* = 6/group).

**Table 11:** Correlations and their significance between individual volatile organic compounds (aldehydes, alkanes, alkynes, alkenes, terpenes, ketones, % from the total volatile compound) in piglet feces and fecal microbiological parameters, pH, dry matter content, and texture hardness.

**Volatile compound**	***r* and *p* values**	**LAB**	**TBC**	**TEC**	**Y and M**	**pH**	**DM**	**Texture**
Nonanal	*r*	−0.253	0.128	0.229	**0.420** ^*^	0.253	0.077	−0.043
	*p*	0.233	0.552	0.281	**0.041**	0.232	0.721	0.840
Decanal	*r*	0.020	0.372	0.131	0.313	0.153	0.162	−0.149
	*p*	0.927	0.073	0.540	0.137	0.475	0.450	0.487
Dodecanal	*r*	−0.150	0.285	0.321	0.339	0.254	0.123	−0.041
	*p*	0.483	0.178	0.126	0.105	0.232	0.566	0.851
Tridecanal <n->	*r*	−0.147	−0.003	**0.458** ^*^	0.299	0.161	−0.074	0.020
	*p*	0.493	0.988	**0.024**	0.155	0.453	0.733	0.926
Pentadecanal	*r*	−0.292	0.107	**0.449** ^*^	0.337	0.328	0.065	0.123
	*p*	0.166	0.618	**0.028**	0.107	0.117	0.764	0.566
Hexadecanal	*r*	−0.339	0.009	0.312	0.270	0.401	−0.085	0.207
	*p*	0.106	0.968	0.138	0.202	0.052	0.692	0.332
*cis*-9-Hexadecenal	*r*	−0.300	0.168	**0.446** ^*^	0.365	0.351	0.075	0.139
	*p*	0.154	0.433	**0.029**	0.080	0.092	0.726	0.518
(2E)-2-Tetradecenal	*r*	0.219	0.237	0.263	0.120	−0.120	0.090	−0.181
	*p*	0.495	0.457	0.410	0.710	0.709	0.782	0.573
Tetradecanal	*r*	0.106	−0.041	0.178	0.128	−0.155	−0.085	−0.109
	*p*	0.623	0.847	0.406	0.552	0.468	0.691	0.611
(Z)-7-Hexadecenal	*r*	0.085	0.150	−0.400	−0.200	−0.127	−0.275	0.028
	*p*	0.693	0.483	0.053	0.349	0.554	0.193	0.896
Octadecanal	*r*	0.082	0.269	−0.380	−0.192	−0.048	−0.290	−0.103
	*p*	0.702	0.205	0.067	0.368	0.824	0.169	0.633
Dodecane	*r*	−0.184	0.216	**0.436** ^*^	**0.437** ^*^	0.227	0.158	0.101
	*p*	0.389	0.310	**0.033**	**0.033**	0.285	0.460	0.638
Tridecane	*r*	−0.217	−0.260	0.028	0.075	0.057	−0.125	−0.004
	*p*	0.308	0.219	0.896	0.727	0.790	0.559	0.986
Tetradecane	*r*	−0.360	−0.041	0.268	**0.440** ^*^	0.333	−0.114	0.127
	*p*	0.084	0.848	0.205	**0.031**	0.111	0.594	0.555
Hexadecane	*r*	0.032	0.018	0.221	0.161	−0.089	−0.014	0.012
	*p*	0.884	0.934	0.300	0.454	0.680	0.947	0.955
Heptadecane	*r*	−0.113	−0.046	0.234	0.246	0.057	−0.021	−0.007
	*p*	0.599	0.830	0.272	0.246	0.790	0.923	0.973
Octadecane	*r*	−0.148	0.086	0.288	0.297	0.100	0.065	0.077
	*p*	0.490	0.688	0.173	0.159	0.641	0.765	0.722
Nonadecane	*r*	0.368	0.152	−0.126	−0.195	**−0.418** ^*^	0.068	−0.093
	*p*	0.077	0.479	0.559	0.362	**0.042**	0.751	0.665
Eicosane	*r*	0.329	0.042	−0.227	−0.239	**−0.410** ^*^	−0.044	−0.061
	*p*	0.117	0.844	0.286	0.260	**0.047**	0.840	0.777
1-octadecyne	*r*	0.235	−0.126	−0.370	−0.242	−0.177	−0.200	−0.109
	*p*	0.270	0.558	0.075	0.254	0.407	0.349	0.613
1-Nonadecene	*r*	−0.097	−0.288	0.146	**−0.475** ^*^	−0.081	−0.193	0.350
	*p*	0.651	0.173	0.497	**0.019**	0.705	0.367	0.094
*β*-Gurjunene	*r*	−0.243	−0.005	**0.455** ^*^	0.242	0.242	−0.009	0.140
	*p*	0.252	0.982	**0.026**	0.255	0.254	0.967	0.515
Methyl octyl ketone	*r*	0.320	−0.054	−0.370	−0.284	−0.330	−0.119	−0.139
	*p*	0.128	0.802	0.075	0.179	0.115	0.581	0.516
Heptylketone	*r*	0.218	−0.161	−0.339	−0.327	−0.200	−0.205	−0.081
	*p*	0.305	0.452	0.105	0.119	0.348	0.337	0.707
Decyl methyl ketone	*r*	0.192	−0.239	**−0.525** ^**^	−0.234	−0.241	−0.272	−0.155
	*p*	0.369	0.261	**0.009**	0.271	0.256	0.199	0.471
Geranyl acetone	*r*	0.088	−0.319	**−0.433** ^*^	−0.269	−0.179	−0.331	0.012
	*p*	0.683	0.128	**0.035**	0.204	0.402	0.114	0.956

LAB–lactic acid bacteria; TBC–total bacteria count; TEC–total enterobacteria count; Y and M–yeast and mold; DM–dry matter. *r*–Pearson Correlation; *p*–significance; ** – correlation is significant at the 0.01 level (two-tailed); * – correlation is significant at the 0.05 level (two-tailed). Correlation interpreted as significant, when *p* ≤ 0.05. Bold values indicate significant correlations (*p* ≤ 0.05).

The VOCs presented in Table 6 (carboxylic acids, fatty acid methyl esters, fatty acids, indole derivatives, and cyclic ethers) did not differ significantly between the control and Lb.-FM groups. Furthermore, diet did not significantly affect the percentage contribution of these compounds to the total VOC profile. However, differences in VOC composition were observed between the two animal groups at the beginning (day 21) and at the end (day 61) of the experiment. At the beginning of the experiment, isohexanoic acid, hexadecanoic acid methyl ester, hexadecanoic acid, and hexadecene epoxide were not detected in the feces of either group of animals. In contrast, at the end of the experiment, 4-methylvaleric acid and hexanoic acid were not detected.

Correlations and their significance between individual carboxylic acids, fatty acid methyl esters, fatty acids, indole derivatives, and cyclic ethers in piglet feces and fecal microbiological parameters, pH, dry matter content, and texture hardness are shown in Table 7. The LAB count in piglet feces showed a moderate positive correlation with hexadecanoic acid methyl ester (*r* = 0.423, *p* = 0.039). The TBC count showed a moderate positive correlation with 4-methylvaleric acid (*r* = 0.438, *p* = 0.032). The TEC count showed moderate positive correlations with acetic acid and propionic acid (*r* = 0.438, p = 0.032 for both), and a moderate negative correlation with hexadecanoic acid (*r* = −0.458, *p* = 0.024). A moderate negative correlation was also found between acetic acid and mold and yeast counts (*r* = −0.518, *p* = 0.010). No significant correlations were observed between individual VOCs and fecal dry matter or texture hardness. However, fecal pH showed moderate negative correlations with 3-methylbutanoic acid, 2-methylbutyric acid, and hexadecanoic acid methyl ester (*r* = −0.435, *p* = 0.034; *r* = −0.434, *p* = 0.034; *r* = −0.477, *p* = 0.018, respectively).

Regarding aromatic hydrocarbons, *α*,*β*-unsaturated aldehydes, phenols, esters, and alcohols in piglet fecal VOC profiles, butylhydroxytoluene showed significant differences between the groups at day 61 (*p* = 0.003) (Table 8). Diet was also identified as a significant factor affecting this compound in feces (*p* < 0.001). At day 61, the concentration of butylhydroxytoluene in the fecal VOC profile of the control group was, on average, 4.16 times higher compared with the Lb.-FM group.

Non-(2E)-enal, butylhydroxytoluene, 3-methylbutanoic acid butyl ester, benzeneacetic acid methyl ester, ethyl hydrocinnamate, Z-2-dodecenol, and cyclododecanol were identified only in the faeces of 61-day-old piglets. In contrast, 1,3-di-tert-butylbenzene, 2,6-di-tert-butyl-4-methylphenol, pentyl isovalerate, 11-dodecen-1-yl acetate, 3-phenylpropanol, and pentadecanol were identified only in the feces of 21-day-old piglets.

Correlations and their significance between individual aromatic hydrocarbons, *α*,*β*-unsaturated aldehydes, phenols, esters, and alcohols in piglet feces and fecal microbiological parameters, pH, dry matter content, and texture hardness are presented in Table 9. No significant correlations were found between the LAB count in piglet feces and the individual VOCs from the above-mentioned groups. The TBC count showed a moderate negative correlation with butylhydroxytoluene (*r* = −0.546, *p* = 0.006). The TEC count showed moderate positive correlations with 2,6-di-tert-butyl-4-methylphenol, 11-dodecen-1-yl acetate, and pentadecanol (*r* = 0.458, *p* = 0.024; *r* = 0.463, *p* = 0.023; and *r* = 0.498, *p* = 0.013, respectively), as well as moderate negative correlations with non-(2E)-enal and butylhydroxytoluene (*r* = −0.474, *p* = 0.019; *r* = −0.518, *p* = 0.010, respectively). A moderate positive correlation was also observed between pentadecanol and yeast and mold counts (*r* = 0.428, *p* = 0.037). No significant correlations were observed between individual VOCs and fecal pH or texture hardness. However, fecal dry matter showed a moderate negative correlation with butylhydroxytoluene (*r* = −0.553, *p* = 0.005).

Regarding aldehydes, alkanes, alkynes, alkenes, terpenes, and ketones in piglet faecal VOC profiles, there were no significant differences between groups at day 21 and 61 (Table 10). Diet was also identified as no significant factor. Although cis-9-hexadecenal was detected in the faeces of 21-day-old piglets, it was not detected at day 61 in either group. In contrast, (2E)-2-tetradecenal, (Z)-7-hexadecenal, octadecanal, nonadecane, eicosane, 1-octadecyne, 1-nonadecene, methyl octyl ketone, heptyl ketone, decyl methyl ketone, and geranyl acetone were detected only in the faeces of 61-day-old piglets. A reduction in some individual VOCs was also observed at day 61 compared with day 21 in fecal samples. Specifically, nonanal showed, on average, 2.03- and 2.15-fold lower concentrations in the control and Lb.-FM groups, respectively. Similarly, dodecanal showed 2.34- and 2.04-fold lower concentrations, pentadecanal showed 6.46- and 3.86-fold lower concentrations, and dodecane showed 3.58- and 3.90-fold lower concentrations in the control and Lb.-FM groups, respectively.

Correlations and their significance between individual aldehydes, alkanes, alkynes, alkenes, terpenes, and ketones in piglet feces and fecal microbiological parameters, pH, dry matter content, and texture hardness are presented in Table 11. No significant correlations were found between the LAB and TBC counts in piglet feces with individual VOCs from the aldehydes, alkanes, alkynes, alkenes, terpenes, and ketones groups. However, the TEC count showed moderate positive correlations with tridecanal, pentadecanal, *cis*-9-hexadecenal, dodecane, and *β*-gurjunene (*r* = 0.458, *p* = 0.024; *r* = 0.449, *p* = 0.028; *r* = 0.446, *p* = 0.029; *r* = 0.436, *p* = 0.033; and *r* = 0.455, *p* = 0.026, respectively), as well as moderate negative correlations with decyl methyl ketone and geranyl acetone (*r* = −0.525, *p* = 0.009; *r* = −0.433, *p* = 0.035, respectively). Moderate positive correlations were also observed between dodecane and tetradecane with yeast and mold counts (*r* = 0.437, *p* = 0.033; *r* = 0.440, *p* = 0.031, respectively). In addition, a moderate negative correlation was observed between 1-nonadecene and yeast and mold counts (*r* = −0.475, *p* = 0.019). Fecal pH showed moderate negative correlations with nonadecane and eicosane (*r* = −0.418, *p* = 0.042; *r* = −0.410, *p* = 0.047, respectively).

## 4 Discussion

Although no significant differences in body weight were observed between the groups, the FCR in the Lb.-FM group was lower, possibly indicating more efficient nutrient utilization compared with the control group. Nevertheless, it can be concluded that the fermented feed supported satisfactory piglet growth. Such improvements may be explained by enhanced nutrient digestibility (43). These effects can be attributed to multiple factors, including the activity of LAB present in fermented feed, which can improve gut microbiota composition and produce enzymes that enhance nutrient utilization. The absence of significant differences in body weight may be related to the experiment’s duration or the piglets’ age, as growth responses to dietary interventions may be less pronounced during early developmental stages (44). It has been reported that improvements in gut health and feed efficiency do not necessarily result in immediate increases in body weight (45).

This study demonstrated that dietary inclusion of Lb.-FM did not adversely affect systemic physiological status, as no significant changes were observed in the blood plasma parameters of piglets, indicating its physiological safety. The observed increase in IgM and IgG concentrations in the blood plasma of 61-day-old piglets in both groups may be associated with the natural maturation of the immune system. Immunoglobulin levels in piglet plasma are known to increase with age as active immunity develops (46). The IgA concentration below the detection limit in the plasma of piglets from both groups may be explained by the fact that IgA responses are more pronounced at the intestinal level rather than in systemic circulation (47). IgA is the predominant antibody class in mucosal secretions and exhibits both anti-inflammatory and pro-inflammatory activities (46). The intestinal mucosal immune system of healthy piglets produces IgA in response to antigen stimulation (48). Furthermore, the increase in ALT activity observed at 61 days of age in both groups may be associated with age-related metabolic changes, as no significant dietary effect on this parameter was detected. ALT is a key indicator of liver function, and moderate increases within physiological ranges may be linked to growth, which is associated with increased metabolic activity. Similar trends were observed for AST activity. The liver plays a central role in immune regulation, which is closely related to growth performance in piglets (49). As the primary organ responsible for metabolism and detoxification, the liver is frequently exposed to circulating antigens, endotoxins, and even microorganisms due to its unique anatomical position (50, 51). Based on the obtained blood plasma results, it can be concluded that the Lb.-FM did not negatively affect liver function or the overall physiological status of the piglets.

Also, the diet did not markedly alter the physicochemical characteristics of feces, as the inclusion of Lb.-FM had no significant effect on fecal pH, dry matter content, or texture hardness in piglets, indicating that the intervention had no measurable effect on these parameters. These findings suggest that the Lb.-FM maintained normal digestive function and gut stability, without disrupting the overall balance of the gastrointestinal environment. The lack of significant differences in fecal pH between the experimental groups may indicate that the buffering capacity of the gastrointestinal tract and microbial activity remained relatively stable regardless of diet. Although fermented feed is often associated with reduced gastrointestinal pH due to the production of organic acids by LAB, such effects are not always reflected in fecal pH, particularly when homeostatic mechanisms regulate intestinal conditions (52, 53). Similarly, the absence of differences in fecal dry matter content suggests that water absorption and digestive efficiency were not significantly influenced by the dietary treatment. Fecal dry matter is commonly used as an indicator of nutrient digestibility and intestinal health, and stable values across treatments imply that both diets supported comparable digestive processes. However, the observed increase in fecal texture hardness in the control group at 61 days of age may indicate differences in intestinal transit time or microbial activity. Softer feces in the Lb.-FM group could be associated with improved gut microbiota balance and enhanced fermentation processes, potentially leading to better water retention and stool consistency. LAB present in fermented feed is known to influence intestinal viscosity and microbial fermentation patterns, which may contribute to these effects. Despite this observation, the overall lack of significant dietary effects suggests that the influence of Lb.-FM on fecal physicochemical properties was limited under the conditions of this study. Similar findings have been reported in previous studies, where probiotic or fermented feed interventions had minimal impact on fecal pH and dry matter, while more pronounced effects were observed in microbial composition and metabolic activity (54).

Also, the dietary treatment indicated a stable gut microbial environment under both feeding conditions and did not markedly alter the overall microbial counts. The absence of significant differences between groups may indicate that the established intestinal microbiota of piglets was relatively resilient to dietary modification, particularly under the experimental conditions and duration applied. It is well recognized that while Lb.-FM and LAB can influence gut microbiota, their effects are often more pronounced at the level of microbial composition and activity rather than viable microbial counts. The observed time-related differences in TBC in the control group may reflect normal microbial succession associated with piglet development. As piglets age, the gastrointestinal microbiota undergoes dynamic changes, including increases in microbial diversity and population density, which are linked to dietary transitions and maturation of the digestive system (55). The lack of a significant dietary effect on microbiological parameters may also be associated with the complexity of gut microbial ecosystems, where interactions among microbial populations can buffer external influences such as feed composition. Additionally, it is possible that the effects of Lb.-FM were more functional than quantitative, influencing microbial metabolism, enzyme activity, or the production of bioactive compounds rather than overall bacterial counts. These findings are consistent with previous studies reporting that probiotic or fermented feed supplementation does not always lead to significant changes in total bacterial counts but may still confer benefits through modulation of microbial balance and gut health (56–60). Therefore, the absence of significant differences in microbiological parameters does not necessarily indicate a lack of biological effect. Overall, the results suggest that Lb.-FM maintains microbiological stability in piglet feces, while potential benefits may be reflected in functional or metabolic changes rather than in absolute microbial counts.

Metataxanomic analysis demonstrated similar microbial composition in piglets’ feces before feeding experiment. Gastrointestinal tract contained normal microbiota with the higher prevalence of anaerobic bacteria from families Prevotellaceae, Lachnospiraceae and Ruminococcaceae. Microorganisms of these families are known as core microbiota of pigs at post weaning phase (61, 62). The prevalence of probiotic *Lactobacillus* and *Faecalibacterium* was slightly higher in the control group but without obvious difference between the groups. At the end of the feeding experiment, numbers of *Lactobacillus* significantly increased in the group fed with fermented feed in comparing with the control group. Although most of the lactobacilli left unidentified up to the species level, overall species variety was wide. Greater abundance of *Faecalibacterium* after experiment in Lb.-FM group also demonstrated positive influence of the fermented feed to microbial changes. The experimental group also had higher numbers of Peptostreptococcaceae, which can be considered a positive effect of fermented feed. Bacteria from this family, particularly *Romboutsia*, are also regarded as probiotic microbiota, along with lactobacilli and *Faecalibacterium* (63–65). The amount of other bacteria overall were similar in both groups and did not significantly differ from the composition which was determined at the beginning of the experiment. The numbers of Enterobacteriaceae and *Campylobacter* significantly decreased at the age of 61 day in both groups and according to previous data, this is normal process of microbial changes during the growth of pigs (66, 67). Finally, fermented feed had an overall positive effect on the gastrointestinal microbial composition of piglets. Although the abundance of probiotic bacteria tended to increase, this difference was not statistically significant, and the overall microbial community structure remained unaffected.

The results indicate that the broad metabolic output of the gut microbiota remained largely stable under the dietary intervention, as the inclusion of Lb.-FM had no significant effect on carboxylic acids, fatty acid methyl esters, fatty acids, indole derivatives, or cyclic ethers in the VOC profile of piglet feces. Similarly, the relative contribution of these compounds to the total VOC profile was not significantly affected by diet. These findings suggest that the Lb.-FM did not markedly alter the overall metabolic output of the gut microbiota at the level of major VOC groups. However, differences in VOC composition between sampling times (day 21 *vs.* day 61) indicate that age had a more pronounced effect than diet. The absence of certain compounds at the beginning of the experiment and the disappearance of others at later stages reflect dynamic shifts in microbial metabolism associated with gastrointestinal development. Such temporal changes are consistent with the maturation of gut microbiota, which is known to influence the production of fermentation-derived metabolites. The observed correlations between individual VOCs and microbiological parameters further support the link between microbial activity and VOC production. Positive correlations between LAB counts and compounds such as hexadecanoic acid methyl ester, as well as between TEC and short-chain fatty acids (e.g., acetic and propionic acids), suggest that these metabolites are closely associated with microbial fermentation processes. In contrast, negative correlations (e.g., between acetic acid and yeast and mold counts) may indicate competitive interactions between microbial groups or differences in substrate utilization. The negative correlations between faecal pH and several branched-chain fatty acids (e.g., 3-methylbutanoic and 2-methylbutyric acids) are in line with the known role of organic acids in lowering intestinal pH. These findings highlight the contribution of microbial fermentation to the physicochemical environment of the gut. Among the identified VOC groups, butylhydroxytoluene was the only VOC significantly affected by diet, with higher concentrations observed in the control group at day 61. This may indicate differences in oxidative processes or microbial degradation pathways influenced by the fermented feed. The lower levels in the Lb.-FM group could suggest a potential protective or stabilizing effect of LAB against the formation or accumulation of this compound. The presence of specific VOCs exclusively at different time points further supports the importance of age-related microbial succession. Compounds detected only at day 61 may be associated with more complex microbial communities and metabolic pathways that develop later in life, while those present only at day 21 may reflect early colonization processes and simpler fermentation patterns. Despite the lack of significant dietary effects on most VOCs, the observed correlations between VOCs and microbial counts indicate that microbial functionality, rather than composition alone, plays a key role in shaping the fecal metabolite profile. This is further supported by the associations between certain aldehydes, hydrocarbons, and ketones with TEC and yeast/mold counts, suggesting that different microbial groups contribute to distinct metabolic pathways. The reduction in concentrations of several aldehydes and hydrocarbons at day 61 compared with day 21 may reflect shifts in substrate availability, microbial activity, or metabolic efficiency as the gut environment matures. These changes may also indicate a transition from primary fermentation products to more stable or further metabolized compounds. Overall, the results suggest that age-related changes in gut microbiota and metabolism play a more significant role than diet in shaping the fecal VOC profile of piglets under the conditions of this study. While fermented feed did not significantly alter the overall VOC composition, it may influence specific metabolic pathways and microbial interactions, as indicated by compound-specific differences and correlations.

This study has several limitations. One limitation is the use of a single pig genetic background and a single fermented feed formulation based on selected *Lactobacillus* spp. Therefore, the observed effects may be specific to this host–microbiota–diet combination, and the findings may not be directly generalizable to other pig breeds or probiotic formulations. Future research incorporating metabolomic profiling of gastrointestinal tract biomarkers may provide a more comprehensive characterization of the effects of fermented feeds. Furthermore, further studies in older animals would help to elucidate the long-term consequences of dietary interventions and thus better understand their long-term effects on growth rate and gut health.

## 5 Conclusion

The inclusion of Lb.-FM in piglet diets did not significantly affect growth performance, blood parameters, fecal physicochemical properties, microbial counts, or fecal VOC profiles, confirming its safety and suitability. However, improved FCR suggests enhanced nutrient utilization. Furthermore, Lb.-FM positively influenced gut microbiota composition by increasing the abundance of probiotic bacteria. Overall, the findings from this study indicate that feeding material fermented with *Lb. plantarum*, *Lb. casei*, *Lb. curvatus*, and *Lb. paracasei* was associated with improved feed conversion ratio (FCR) parameters. Although some positive trends in the abundance of beneficial bacteria were observed, these differences were not statistically significant, also meaning that microbial homeostasis wasn’t disrupted. Therefore, the results suggest that fermented feed may contribute to sustainable nutritional strategy for pig production.

## Data Availability

The original contributions presented in the study are included in the article, further inquiries can be directed to the corresponding author/s. Sequences were deposited at the NCBI database by the access number PRJNA1441863.
